# Sensor-cable-probe and sampler for early detection and prediction of dry matter loss and real-time corn grain quality in transport and storage

**DOI:** 10.1038/s41598-023-32684-4

**Published:** 2023-04-07

**Authors:** Camila Fontoura Nunes, Paulo Carteri Coradi, Lanes Beatriz Acosta Jaques, Larissa Pereira Ribeiro Teodoro, Paulo Eduardo Teodoro

**Affiliations:** 1 Department of Agricultural Engineering, Rural Sciences Center, Federal University of Santa Maria, Avenue Roraima, 1000, Camobi, Santa Maria, Rio Grande do Sul 97105-900 Brazil; 2 Department of Agricultural Engineering, Laboratory of Postharvest (LAPOS), Campus Cachoeira do Sul, Federal University of Santa Maria, Highway Taufik Germano, 3013, Passo D’Areia, Cachoeira do Sul, Rio Grande do Sul 96506-322 Brazil; 3 Department of Agronomy, Campus de Chapadão do Sul, Federal University of Mato Grosso do Sul, Chapadão do Sul, Mato Grosso do Sul 79560-000 Brazil

**Keywords:** Sustainability, Information technology

## Abstract

Taking into account that the transport of grains can be carried out over long distances and that the mass of grains during transport often has high moisture content, there may be risks of heat and moisture transfer and heating of the grains mass, proving quanti-qualitative losses. Thus, this study aimed to validate a method with probe system for real-time monitoring of temperature, relative humidity and carbon dioxide in the grain mass of corn during transport and storage to detect early dry matter losses and predict possible changes on the grain physical quality. The equipment consisted of a microcontroller, system's hardware, digital sensors to detect air temperature and relative humidity, a non-destructive infrared sensor to detect CO_2_ concentration. Real-time monitoring system determined early and satisfactorily in an indirect way the changes in the physical quality of the grains confirming by the physical analyses of electrical conductivity and germination. The equipment in real-time monitoring and the application of Machine Learning was effective to predict dry matter loss, due to the high equilibrium moisture content and respiration of the grain mass on the 2-h period. All machine learning models, except support vector machine, obtained satisfactory results, equaling the multiple linear regression analysis.

## Introduction

Despite the high corn grain production, it is verified that there are great losses in the post-harvest stages due to the precarious transport, facilities, and handling of the grain receiving, drying, and storage operations. During grain transportation, it is estimated that there are losses of 0.25% per ton of grain transported^1^. Losses in road transport occur due to poor roads, vehicle speed, deteriorated truck bodies, among others^2^. Furthermore, once the grains are harvested, they remain biologically active and depending on the conditions in which they are found can trigger several metabolic reactions causing both quantitative and qualitative losses^3^.

Among the factors influencing grain quality at postharvest are high grain moisture content, temperature, and intergranular relative humidity^4,5^. Elevation of these parameters can increase grain respiration and metabolic activity, causing deteriorations in the physicochemical quality of the grain and dry matter consumption, insect proliferation, and fungal infection in the grain mass^6,7^.

Taking into consideration that often the grain transport is carried out over long distances, and that the grain mass transported may have moisture content above the optimal conditions for storage, the risks of moisture and heat transfer during transport are high, causing possible grain mass heating^8–10^.

Thus, to avoid these problems in transport and possible potential factors of alterations in the following post-harvest processes, it is important to perform real-time monitoring of the temperature and relative humidity of the intergranular air in order to estimate the equilibrium moisture content, as well as to monitor the levels of carbon dioxide and the respiratory intensity of the grain mass over the transport^11,12^. From this information, it is possible to detect early and predict changes in grain quality^13^.

Several studies on quantitative and qualitative postharvest losses have been carried out^7,10^. However, there are few studies addressing the qualitative losses due to metabolic activity in the transport process as a possible influencer for the triggering and intensification of losses across the subsequent post-harvest operations.

Thus, real-time monitoring of corn grain mass during transportation and the employment of predictive algorithms could help in the early detection and prediction of possible quantitative and qualitative losses of corn grains. Thus, this study aimed to validate a non-destructive technological system for real-time monitoring of temperature, relative humidity, and carbon dioxide in corn grain mass during transportation and storage as a function of different initial moisture content, in order to detect early losses of dry matter using Machine Learning algorithms and predict possible changes in the physical quality of the grains.

## Material and methods

### Hardware and software design

**Figure 1:**
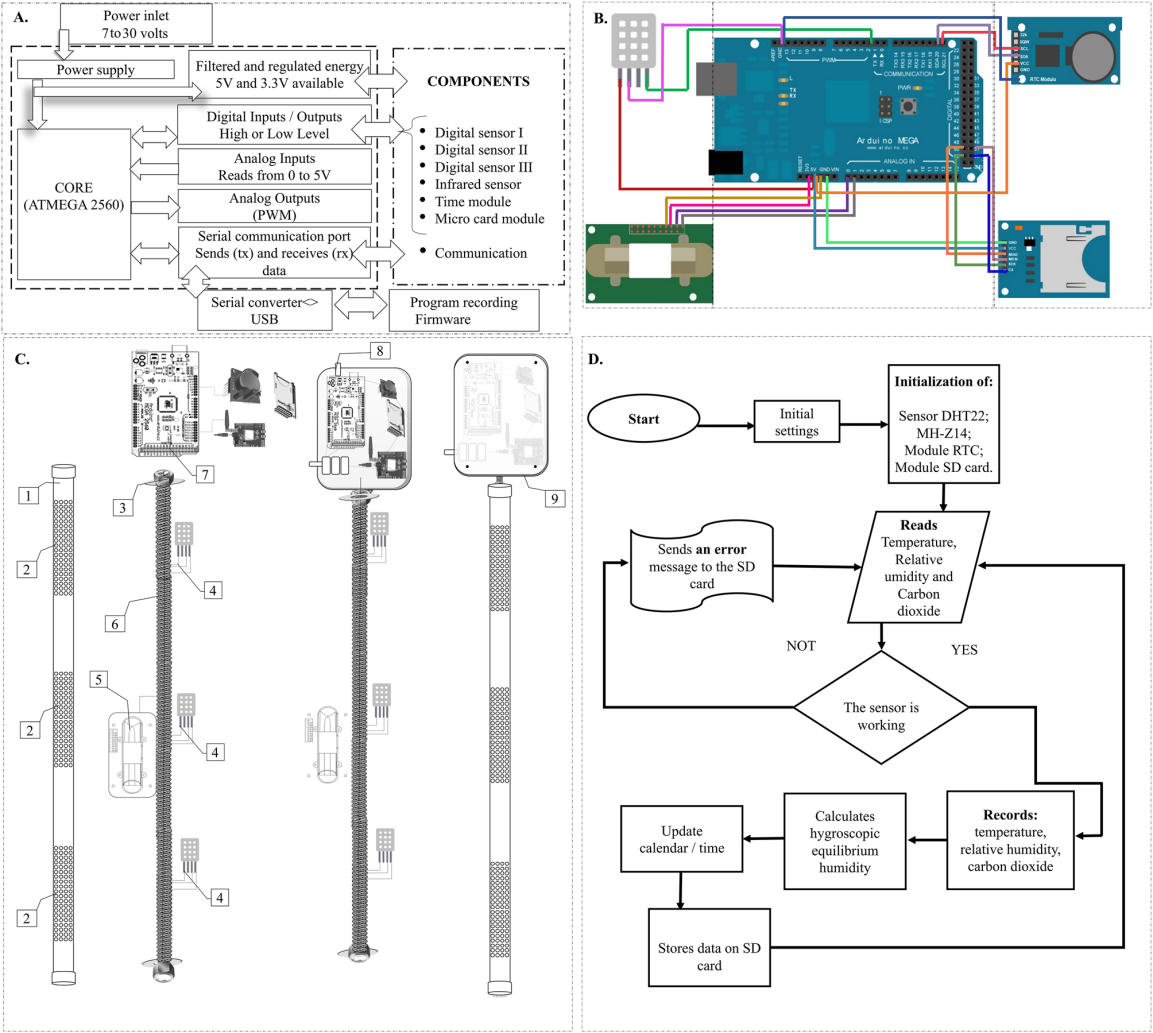
Block diagram of the monitoring operating system (**A**). Connection of the components to the microcontroller using jumper cables (**B**). Structural part of the probe for application in the grain mass (**C**), where: 1- polyvinyl chloride tube, 2—holes for air entry, 3—fixing elements, 4—DHT22 sensors, 5—MH-Z14 sensor, 6—threaded bar and support cabling, 7—equipment hardware, 8—internal view of the probe, 9—probe for application in the grain mass. Flowchart of the programming used for the operation of the monitoring system (**D**).

For monitoring the grain mass during road transport, a portable device has been developed^14^. The device consists of an Arduino Mega 2560 microcontroller (model Mega 2560, Arduino LLC, Italy) as the control core. The system hardware includes three digital sensors to detect air temperature and relative humidity (model DHT22, Aosong Electronics, Guangzhou, China), a non-destructive infrared sensor to detect CO_2_ concentration (model MHZ-14, Winsen, China), real-time clock modules (model DS3231, flip-flop, China), and a micro-SD card (model Greatzt, import, China). The block diagram of the control system is shown in Fig. 1A. Output data from the digital sensor, infrared sensor and modules are connected to the I/O communication terminals of the microcontroller which are responsible for physical communication, component integration, and data calculation. The electrical connection diagram of each component via jumper cables is shown in Fig. 1B.

Temperature and relative humidity sensors (model DHT22, Aosong Electronics, Guangzhou, China) were attached to three ends of a threaded bar and the CO_2_ sensor (model MHZ-14, Winsen, China) was attached to the central part. The real-time clock module (model DS3231, flip-flop, China) and the micro-SD card (model Greatzt, Import, China) were packed in a plastic box (Patola Electroplastics Ind. Com. Ltda, Brazil). Figure 1C shows the structure of the equipment with the sensors arranged along the threaded bar and protected by a polyvinyl chloride probe. The equipment has its own power supply with three series-connected batteries.

The software used on the Arduino board was programmed using the C++ language, with most of the libraries provided by the platform (S1). Arduino IDE (Integrated Development Environment) was used to develop the embedded firmware for the Atmega 2560 microcontrollers. The flowchart shown in Fig. 1D refers to the operation program of the equipment used to monitor the grain mass during road transport^15^.

To ensure the same condition for the evaluations, the temperature and relative humidity sensors (model DHT22, Aosong Electronics, Guangzhou, China) were heated to a temperature of 24 °C. With an initial temperature of 24 °C, the sensors operated for 40 min and their measurement was recorded every minute. Using the infrared sensor (model MHZ-14, Winsen, China), the initial calibration was performed on the Arduino itself and the CO_2_ concentration was recorded^16^.

### Setting the probe hole diameter

To define the hole diameter, the temperature, relative humidity and carbon dioxide sensors were placed in probes with different hole diameters (7.5, 7.0 and 6.5 mm), drilling heights (470, 235 and 117.5 mm) and grain moisture content (12 and 16%). The holes were drilled to allow air to enter and facilitate the sensors’ response. For setting the best fitting probe diameter and drilling height, one of the requirements was that they meet the two moisture content analyzed.

After defining the hole diameter and drilling height that best suited the corn kernels, at different moisture content, the equipment was validated in the laboratory. For this purpose, temperature and relative humidity sensors were placed in the probe at the higher, center, and lower positions, while the carbon dioxide sensor was placed in the center position.

To perform the monitoring of the above-mentioned variables in the grain mass, the probe with the sensors was placed into a box built with plywood material (dimensions 0.2 × 0.2 × 1.8 m), simulating the grain load profile in a transportation system. Sensor readings were taken until the values of temperature, relative humidity and carbon dioxide concentration were stabilized.

### Probe development and construction

Probe was constructed using a polyvinyl chloride tube with 50 mm diameter and 1500 mm height, with three drilled regions (higher, center and lower), with holes with 6.5 mm diameter and 235 mm drilling height. Setting the diameter of the holes and the height of the probe drilling were given based on the shortest response time that the sensors had for the measured variables (temperature, relative humidity, and CO_2_) in the intergranular air. Figure 1C shows the distribution of the holes at the higher, center and lower of the probe.

**Figure 2:**
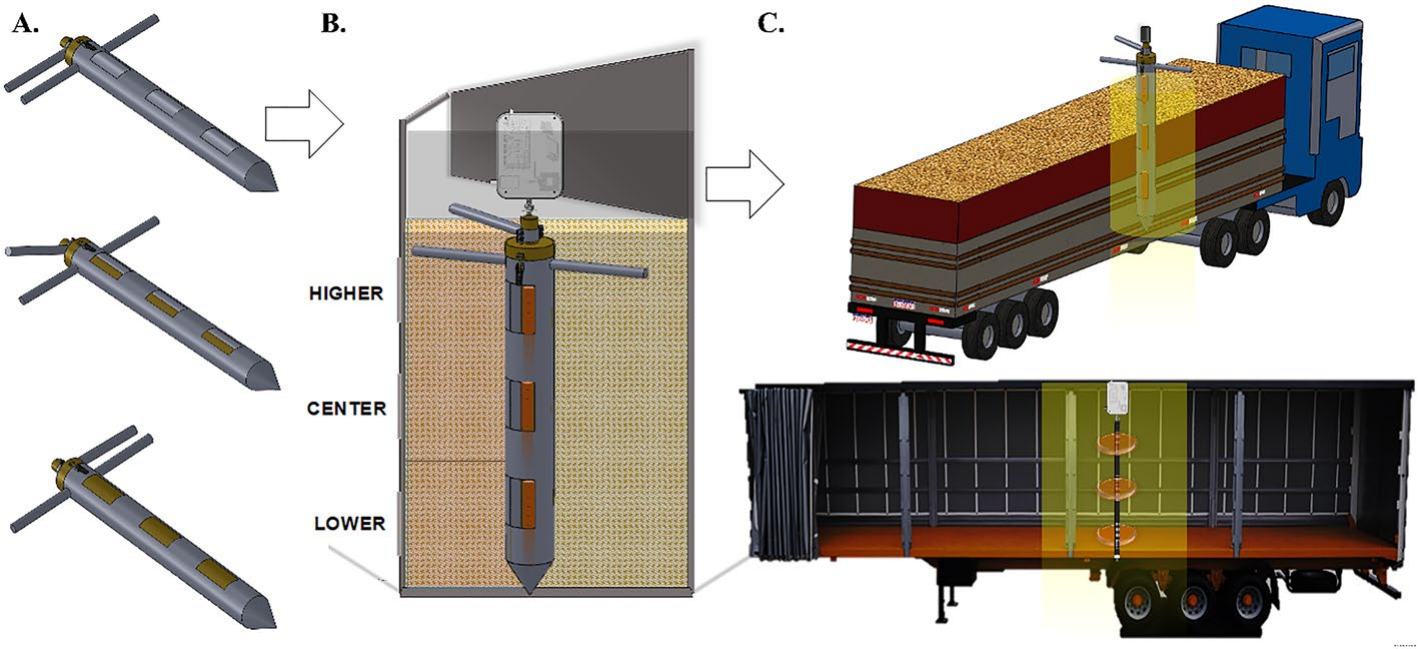
Grain sampling tube (**A**). Representation of the grain mass profile (higher, center and lower positions) and the monitoring system (**B**). Representation of the monitoring system and grain transportation (**C**).

A metal grain sampling tube was developed to couple to the probe (Fig. 2A). The tube consisted of two overlapping tubes, with a tip at the lower position and a swivel arm at the higher position. This enabled the probe to be protected and increased the accuracy of the intergranular grain reading. Furthermore, it allowed for sampling in the different layers of the grain lot profile. The tube contained openings/cells at the higher, center and lower along its length, as shown in Fig. 2B.

### Real-time monitoring of grain mass quality

The metal sampler and the probe with the sensors were inside the grain mass. The variables temperature, relative humidity, and carbon dioxide in the intergranular grain mass were monitored in real-time at intervals of 1.87 s per response for 24 h, simulating the transport conditions (Figs. 2B,C). Filling of each load was performed using grains with 12 and 14% moisture content. Samples were collected at 0, 120, 480, and 1440 min of transport at the three positions (higher, center and lower) of the load. The variation in the equilibrium moisture content was calculated using a modified version of Henderson’s equation (Eq. 1)^17^.

1 $$ EMC = \left\{ {{ln(1} - a_{w} {)/}\left[ { - a{(T} + b{)}} \right]} \right\}^{1/c} $$

where *EMC*—equilibrium moisture content (°C), *a*_*w*_—water activity (decimal), *T*—air temperature (°C), *a*, *b*, *c*—corn parameters (*a* = 8.6541 × 10^–5^, *b* = 49.810, *c* = 1.8634).

To determine the loss of dry matter (LDM) over time, an estimation model was used regarding the CO_2_ concentration (Eq. 2)^18^.

2 $$ LDM = {100(CO}_{{2}} - {O}_{{2}} {)}\left( {\frac{{\varepsilon \rho {U}}}{{{2}\rho ({1} - {U)RT}}}} \right) $$

where *LDM*—loss of dry matter (%), *O*_*2*_—oxygen concentration (21%), *CO*_2_—carbon dioxide concentration measured in the grain mass (%), *Ɛ* – intergranular porosity (%), *P*—local atmospheric pressure (96 kPa), *W*_*g*_—molar mass of glucose (180 kg kmol^−1^), *ρ*—apparent specific mass of grains (kg m^−3^), *U*—grain moisture content (decimal), *R*—constante dos gases perfeitos (8.314 kJ kmol^−1^ K^−1^), T—temperatura intergranular (K).

### Corn quality analysis

Moisture content was determined by drying in a forced air circulation oven at a temperature of 105 ± 1 °C for 24 h in four replicates^19^. The apparent specific mass was obtained by weighing a mass of grains placed in a known volume^19^. Four replicates were performed for each treatment. Electrical conductivity was determined according to ISTA^20^ methodology. Four replicates with 50 grains for each treatment were counted and weighed. Samples were placed in plastic cups containing 75 mL of distilled water and placed in a temperature-controlled chamber at 25 °C for 24 h. The electrical conductivity was obtained by conductivity meter and the result was expressed in µS cm^−1^ g^−1^. Germination test was performed in four experimental units with four subsamples of 50 grains for each treatment. Grains were placed on germitest paper, which was soaked in distilled water. The amount of distilled water was 2.5 times the dry substrate mass. Paper rolls were stored in a Mangelsdorf germinator at 25 °C. Counting was performed on the seventh day after sowing and the results were expressed in percentage^19^.

### Multivariate statistical analysis

Firstly, a principal component analysis (PCA) was performed along with k-means clustering, which clusters treatments whose centroids are closest until there is no significant variation in the minimum distance of each observation to each of the centroids. Next, to verify the interrelationship between variables and treatments, a canonical variable analysis (CA) was performed. This technique is similar to PCA but allows the residual variation between replicates of the same treatment to be taken into account. Afterward, Pearson's correlation coefficients were estimated to verify the association between variables in processing conditions. A correlation network was constructed to graphically express the results. In this procedure, green lines link variables with positive correlation while red lines link negatively correlated variables. The thickness of the line is proportional to the correlation magnitude. These analyses were performed using the *ggfortify* package from the R software and followed the recommended procedures by Naldi et al.^21^ (Table 1).

**Table 1:** Multivariate statistics as a function of initial grain water content, position/layer and grain monitoring time.

Moisture content (% w.b.)	Position	Time (min)	Clusters
12	Lower	0	1
12	Lower	60	2
12	Lower	480	3
12	Lower	1440	4
12	Center	0	5
12	Center	60	6
12	Center	480	7
12	Center	1440	8
12	Higher	0	9
12	Higher	60	10
12	Higher	480	11
12	Higher	1440	12
16	Lower	0	13
16	Lower	60	14
16	Lower	480	15
16	Lower	1440	16
16	Center	0	17
16	Center	60	18
16	Center	480	19
16	Center	1440	20
16	Higher	0	21
16	Higher	60	22
16	Higher	480	23
16	Higher	1440	24

### Machine learning models

Machine learning (ML) models tested were: artificial neural network (ANN), the decision tree algorithms M5P and REPTree, random forest (RF), and support vector machine (SVM). A multiple linear regression (LR) was used as the control model. The ANN tested consists of a multilayer perceptron with a single hidden layer formed by a number of neurons equal to the number of attributes plus the number of classes, all divided by 2^22^.

M5P model is a reconstruction of Quinlan’s M5 algorithm, which is based on the conventional decision tree with the addition of a linear regression function to the leaf nodes^23^. M5P is an adaptation of the C4.5 classifier that can be used in regression problems with an additional pruning step based on an error reduction strategy. REPTree model uses decision tree logic and creates several trees at different repetitions. It then selects the best tree using information gain and performs error reduction pruning as the splitting criterion^24^. RF model is able to produce multiple prediction trees for the same dataset and uses a voting scheme among all these learned trees to predict new values^25^.

The prediction of the variables apparent specific mass (ASM), electrical conductivity (EC), germination (GERM), and loss of dry matter (LDM) of corn was performed by LR and ML models in a tenfold stratified randomized cross-validation with 10 repetitions (100 runs for each model). The input variables were: grain moisture content, monitoring time, intergranular temperature and relative humidity, equilibrium moisture content, and carbon dioxide concentration. The statistics used to verify the quality of the fit were Pearson's correlation coefficient (r) between the observed values and those predicted by each model and the mean absolute error (MAE) of the predicted values in relation to the observed ones. Machine learning analyses were performed on Weka 3.9.4 software using the default configuration for all models tested^26^. All analyses were performed on an Intel^®^ CoreTM i5 CPU with 6 GB of RAM.

After obtaining the r and MAE statistics, analysis of variance was performed considering an entirely randomized design with 10 repetitions (folds). Means were grouped by the Scott–Knott test at 5% probability. Bar graphs were constructed for each variable (r and MAE) considering the models and inputs tested. These analyses were performed on R software^27^ using the packages *ExpDes.pt* and *ggplot2*.

### Ethical statements

Te experimental research and feld studies on plants and plant material were comply with local and national regulations. Te study complied with institutional, national, and international guidelines and legislation. Te authors complied with the IUCN Policy Statement on Research Involving Species at Risk of Extinction and the Convention on the Trade in Endangered Species of Wild Fauna and Flora for the collection of plant or seed specimens. Te authors declare that no wild plants were collected and/or used in this scientifc work.

## Results and discussion

### Validation of the corn grain mass monitoring system

**Figure 3:**
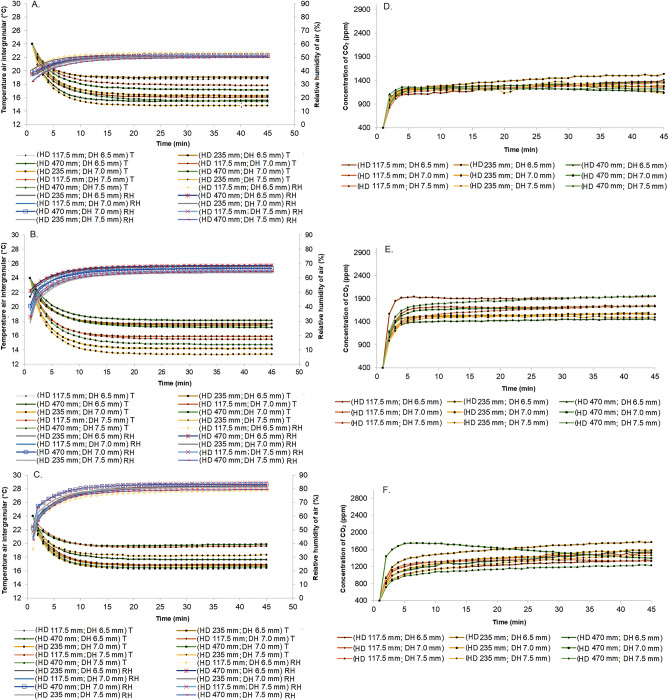
Temperature and relative humidity of corn grain mass with 12% (**A**), 16% (**B**), 25% (**C**) moisture contents in tube with holes of 6.5, 7.0 and 7.5 mm and drilling heights of 117.5, 235 and 470 mm. CO_2_ concentration of corn grain mass with 12% (**D**), 16% (**E**), 25% (**F**) moisture contents in tube with holes of 6.5, 7.0 and 7.5 mm and drilling heights of 117.5, 235 and 470 mm.

Figures 3A–F show the curves of temperature, relative humidity, and intergranular CO_2_ diffusion using real-time monitoring by sensors as a function of probe diameter and drilling height for corn grains with initial moisture contents of 12, 16, and 25%. (w.b.). The behavior of the curves was similar within each monitored variable (Tables 2, 3, 4). After calibration of the sensors at time zero, there was a reduction in temperature and an increase in relative humidity and intergranular CO_2_, with a tendency for the curves to stabilize over time. These findings demonstrate the consistency of the monitored variables, as well as the accuracy and functionality of the monitoring system^28^.

**Table 2:** Polynomial regression equations for air temperature (°C), air relative humidity (%) and CO_2_ (ppm) of corn grain mass at 12% moisture content in tubes with different hole diameters and heights drilling.

Diameter (mm)	Height (mm)	Air temperature (°C)	Air relative humidity (%)	CO_2_ (ppm)
6.50	117.5	y = 0.0042x^2^–0.2421x + 21.698	y = − 0.0104x^2^ + 0.6227x + 45.537	y = − 0.2055x^2^ + 16.43x + 1010.2
		R^2^ = 0.7101	R^2^ = 0.7865	R^2^ = 0.455
	235	y = 0.0038x^2^–0.2237x + 22.19	y = − 0.009x^2^ + 0.531x + 44.698	y = − 0.4124x^2^ + 30.762x + 957.54
		R^2^ = 0.7357	R^2^ = 0.7907	R^2^ = 0.7311
	470	y = 0.0064x^2^–0.367x + 19.878	y = − 0.0121x^2^ + 0.7354x + 40.182	y = − 0.3478x^2^ + 16.932x + 1055.8
		R^2^ = 0.6618	R^2^ = 0.858	R^2^ = 0.1734
7.00	117.5	y = 0.006x^2^–0.3485x + 20.465	y = − 0.0119x^2^ + 0.7216x + 40.386	y = − 0.4499x^2^ + 25.924x + 898.65
		R^2^ = 0.7028	R^2^ = 0.8101	R^2^ = 0.5057
	235	y = 0.0062x^2^–0.3731x + 21.131	y = − 0.0126x^2^ + 0.7835x + 39.987	y = − 0.4564x^2^ + 24.401x + 972.8
		R^2^ = 0.7853	R^2^ = 0.8695	R^2^ = 0.3728
	470	y = 0.0053x^2^–0.3171x + 21.447	y = − 0.0116x^2^ + 0.7131x + 41.363	y = − 0.3897x^2^ + 25.125x + 919.2
		R^2^ = 0,7817	R^2^ = 0.8598	R^2^ = 0.5648
7.50	117.5	y = 0.005x^2^–0.2966x + 21.589	y = − 0.0108x^2^ + 0.6598x + 42.34	y = − 0.4528x^2^ + 29.593x + 893.53
		R^2^ = 0.7745	R^2^ = 0.8398	R^2^ = 0.6505
	235	y = 0.0072 × ^2^–0.4182x + 19.691	y = − 0.016x^2^ + 0.9557x + 37.285	y = − 0.6774x^2^ + 39.083x + 880.12
		R^2^ = 0.6918	R^2^ = 0.8074	R^2^ = 0.5077
	470	y = 0.0064x^2^–0.3968x + 21.395	y = − 0.0141x^2^ + 0.8909x + 36.282	y = − 0.2451x^2^ + 12.394x + 1009.7
		R^2^ = 0.8487	R^2^ = 0.8786	R^2^ = 0.1249

**Table 3:** Polynomial regression equations for air temperature (°C), air relative humidity (%) and CO_2_ (ppm) of corn grain mass at 16% moisture content in tubes with different hole diameters and heights drilling.

Diameter (mm)	Height (mm)	Air temperature (°C)	Air relative humidity (%)	CO_2_ (ppm)
6.50	117.5	y = 0.0045x^2^–0.2653x + 21.493	y = − 0.0161x^2^ + 0.9772x + 55.093	y = − 0.454x^2^ + 27.799x + 1164.1
		R^2^ = 0.7415	R^2^ = 0.755	R^2^ = 0.4127
	235	y = 0.0051x^2^–0.295x + 21.008	y = − 0.0153x^2^ + 0.9169x + 55.463	y = − 0.3791x^2^ + 26.294x + 1369.3
		R^2^ = 0.7071	R^2^ = 0.7878	R^2^ = 0.3708
	470	y = 0.0047x^2^–0.2792x + 21.998	y = − 0.013x^2^ + 0.8016x + 56.275	y = − 0.2942x^2^ + 16.315x + 1066.2
		R^2^ = 0.7966	R^2^ = 0.8499	R^2^ = 0.1942
7.00	117.5	y = 0.0068x^2^–0.385x + 20.947	y = − 0.0242x^2^ + 1.4136x + 47.85	y = − 0.8854x^2^ + 52.732x + 1460.5
		R^2^ = 0.6735	R^2^ = 0.7067	R^2^ = 0.4686
	235	y = 0.0075x^2^–0.4407x + 19.852	y = − 0.025x^2^ + 1.4879x + 45.055	y = − 0.4766x^2^ + 24.182x + 1041
		R^2^ = 0.7256	R^2^ = 0.6904	R^2^ = 0.2838
	470	y = 0.006x^2^–0.36x + 21.367	y = − 0.0198x^2^ + 1.2073x + 50.255	y = − 0.6805x^2^ + 41.679x + 1141.2
		R^2^ = 0.7781	R^2^ = 0.7737	R^2^ = 0.5529
7.50	117.5	y = 0.0064x^2^–0.3782x + 20.494	y = − 0.0209x^2^ + 1.2672x + 46.103	y = − 0.6413x^2^ + 38.939x + 1181.8
		R^2^ = 0.7426	R^2^ = 0.7413	R^2^ = 0.524
	235	y = 0.008x^2^–0.4611x + 18.615	y = − 0.0264x^2^ + 1.5714x + 43.178	y = − 0.7152x^2^ + 39.16x + 1311.1
		R^2^ = 0.6587	R^2^ = 0.7217	R^2^ = 0.3836
	470	y = 0.0072x^2^–0.4279x + 20.138	y = − 0.022x^2^ + 1.3312x + 45.271	y = − 0.6364x^2^ + 39.086x + 1100.6
		R^2^ = 0.7434	R^2^ = 0.7514	R^2^ = 0.5648

**Table 4:** Polynomial regression equations for air temperature (°C), air relative humidity (%) and CO_2_ (ppm) of corn grain mass at 25% moisture content in tubes with different hole diameters and heights drilling.

Diameter (mm)	Height (mm)	Air temperature (°C)	Air relative humidity (%)	CO_2_ (ppm)
6.50	117.5	y = 0.0057x^2^–0.3279x + 20.397	y = − 0.0251x^2^ + 1.5569x + 55.705	y = − 0.3166x^2^ + 22.135x + 735.4
		R^2^ = 0.6897	R^2^ = 0.7079	R^2^ = 0.733
	235	y = 0.0054x^2^–0.3137x + 20.842	y = − 0.025x^2^ + 1.5197x + 60.321	y = − 0.5353x^2^ + 43.302x + 762,8
		R^2^ = 0.7032	R^2^ = 0.7657	R^2^ = 0.9207
	470	y = 0.005x^2^–0.2995x + 21.416	y = − 0.0234x^2^ + 1.4601x + 60.562	y = − 0.5713x^2^ + 43.546x + 945.43
		R^2^ = 0.7611	R^2^ = 0.8215	R^2^ = 0.8332
7.00	117.5	y = 0.0056x^2^–0.3252x + 20.715	y = − 0.0262x^2^ + 15.868x + 59.2	y = − 0.4464x^2^ + 35.641x + 734.31
		R^2^ = 0.708	R^2^ = 0.7465	R^2^ = 0.906
	235	y = 0.0053x^2^–0.3035x + 20.185	y = − 0.0262x^2^ + 1.5847x + 59.363	y = − 0.4258x^2^ + 31.156x + 807.9
		R^2^ = 0.6212	R^2^ = 0.7358	R^2^ = 0.7963
	470	y = 0.0031x^2^–0.1651x + 21.566	y = − 0.019x^2^ + 1.1229x + 67.133	y = − 0.6544x^2^ + 26.248x + 1585.6
		R^2^ = 0.5492	R^2^ = 0.7058	R^2^ = 0.2282
7.50	117.5	y = 0.0039x^2^–0.2252x + 22.084	y = − 0.0217x^2^ + 1.3369x + 65.302	y = − 0.5253x^2^ + 30.33x + 810.73
		R^2^ = 0.7331	R^2^ = 0.7813	R^2^ = 0.6695
	235	y = 0.0033x^2^–0.1993x + 22.574	y = − 0.017x^2^ + 1.0582x + 68.774	y = − 0.5376x^2^ + 40.527x + 844.38
		R^2^ = 0.8313	R^2^ = 0.7985	R^2^ = 0.8749
	470	y = 0.0056x^2^–0.3199x + 20.438	y = − 0.0242x^2^ + 1.4558x + 59.215	y = − 0.3491x^2^ + 28.951x + 637.96
		R^2^ = 0.6558	R^2^ = 0.7429	R^2^ = 0.9234

Among the curves, a variation of the monitored variables in function of the initial moisture content of the grains, diameter and drilling height of the probe, and monitoring time was observed. There was a difference of around 4 °C between the temperature curves, 5% in relative humidity, and 500 ppm of CO_2_ from the stabilization of the curves. The largest variations of temperature, relative humidity, and CO_2_ occurred in the grain mass with 16 and 25% moisture content throughout the monitoring time. Intergranular temperature and relative humidity sensors took about 10 min to stabilize, while the CO_2_ sensor needed about 5 min to reach an equilibrium in the diffusion process. Zhang et al.^5^ evaluated the CO_2_ concentration at various points in the mass of stored grain. The authors found that CO_2_ concentration was sensitively detected at a horizontal distance of 2 m from the hot spot, and 1 m from the hot spot in the vertical direction. According to the authors, the method of detecting CO_2_ concentration at multiple fixed points helped to more accurately quantify grain deterioration.

Huang et al.^10^ evaluated the effective diffusion coefficient of carbon dioxide (CO_2_) through bulk corn grain mass at temperatures of 10, 20, and 30 °C and grain moisture contents of 14.0, 18.8, and 22.2% (w.b). The authors found that the respiration rate of corn increased with increasing grain temperature and moisture content. As the respiration rate increased, it had a greater effect on the diffusion pattern when measuring the effective CO_2_ diffusion coefficient. The effective CO_2_ diffusion coefficients ranged from 3.10 × 10^–6^ to 3.93 × 10^–6^ m^2^ s^−1^.

Among the hole diameters and drilling heights of the probe, we found that 6.5 mm and 235 mm, respectively, achieved the best fit and stabilization of the temperature, relative humidity, and CO_2_ monitoring curves over time (Tables 2, 3, 4). In general, the R^2^ values were low, which is justified by the scale of application, which was close to a real condition in grain volume.

### Monitoring corn grain mass during transport

After the equipment validation, it was applied to a real scale analysis, where a grain transportation system was simulated. In this experiment, the variables temperature, relative humidity, and CO_2_ were monitored to determine the indirect physical quality of the grains with moisture contents of 12 and 16% in three vertical positions of the grain mass profile (higher, center and lower) over 0, 2, 8, and 24 h.

**Figure 4:**
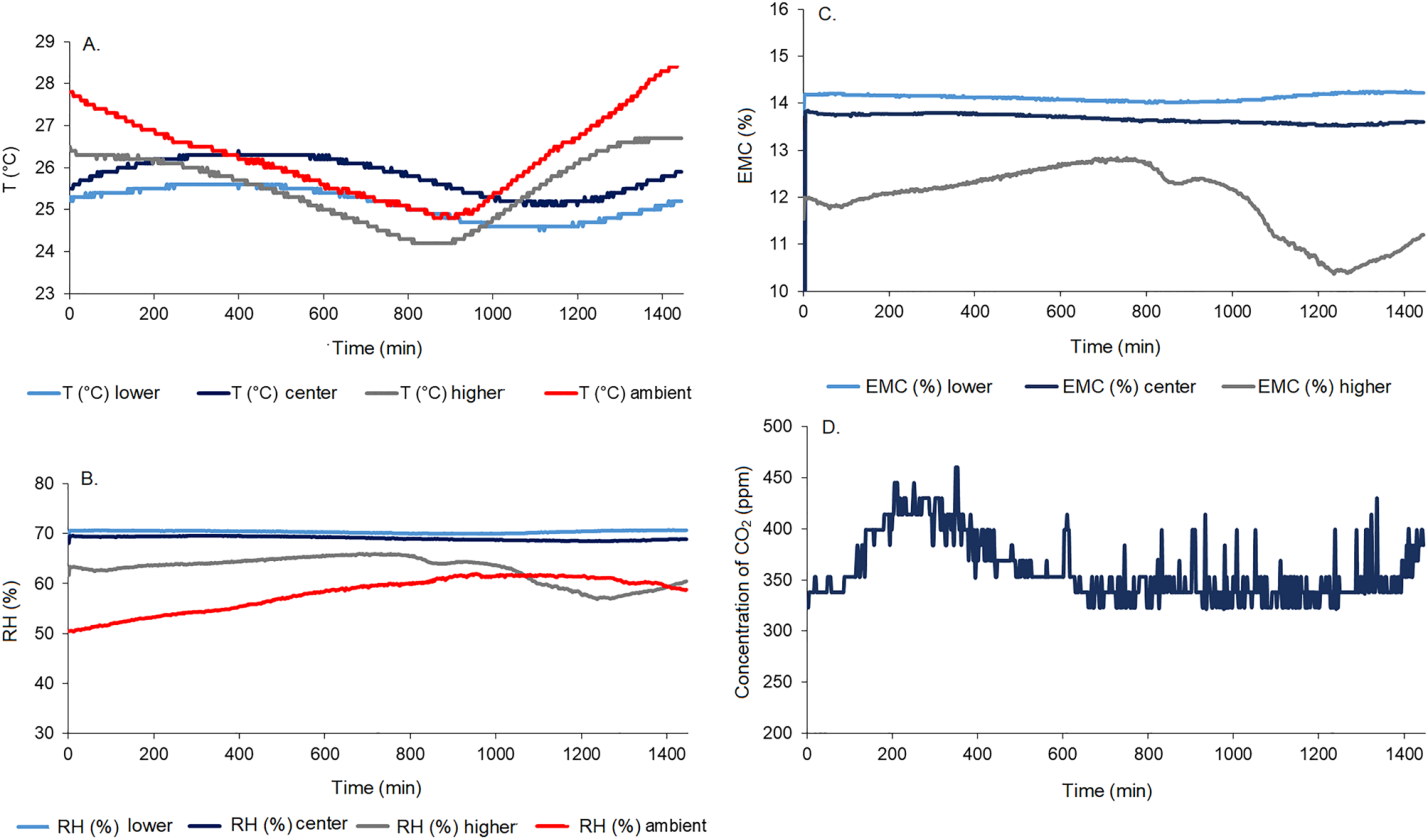
Monitoring temperature (**A**), relative humidity (**B**), equilibrium moisture content (**C**), carbon dioxide (**D**) of the intergranular air in the corn grain mass at 12% moisture content (w.b.) over time.

By Fig. 4A it can be seen that the grain mass with moisture contents at 12% obtained an increase in temperature in the lower and center layer in the first 8 h of monitoring, and subsequently a decrease in temperature until 24 h of monitoring. Differently occurred for the higher position of the grain layer, when a decrease in temperature was observed in the first hours (until 10 h) and an increase in temperature until 24 h of monitoring. These results influenced the intergranular relative humidity (Fig. 4B), which was constant from the beginning to the end of the monitoring time in the lower and center positions but showed an oscillation with a slight reduction after 10 h of monitoring.

Temperature and relative humidity results indicated a constant condition of 14.2 and 13.9% equilibrium moisture content of the grain mass in the lower and center positions, respectively, throughout the monitoring time (Fig. 4C). On the other hand, following the variations observed in Figs. 4A,B, the equilibrium moisture content of the grains in the higher layer increased from 12 to 13% by 10 h of monitoring, subsequently reduced to 10.4% at 12 h, reaching a condition of 11.2% by the end of the monitoring time. Changes observed in the intergranular condition, especially in the higher layer were also influenced by the variation occurring with the temperature and relative humidity of the environment (Fig. 4A,B).

Bakhtavar et al.^29^ evaluated the water adsorption on wheat, corn, cotton and quinoa grains packed in airtight bags and traditional packaging materials, including paper, polypropylene, jute and cloth bags, in environments with 60, 70, 80 and 90% relative humidity. The authors observed that the moisture contents of the grain increased in the traditional packaging materials with increasing relative humidity. According to the authors, the storage of the grains in hermetic bags reduced the respiration of the mass of grains and the variation of the moisture contents, collaborating to the maintenance of the equilibrium moisture content of the grains in safe storage conditions.

**Figure 5:**
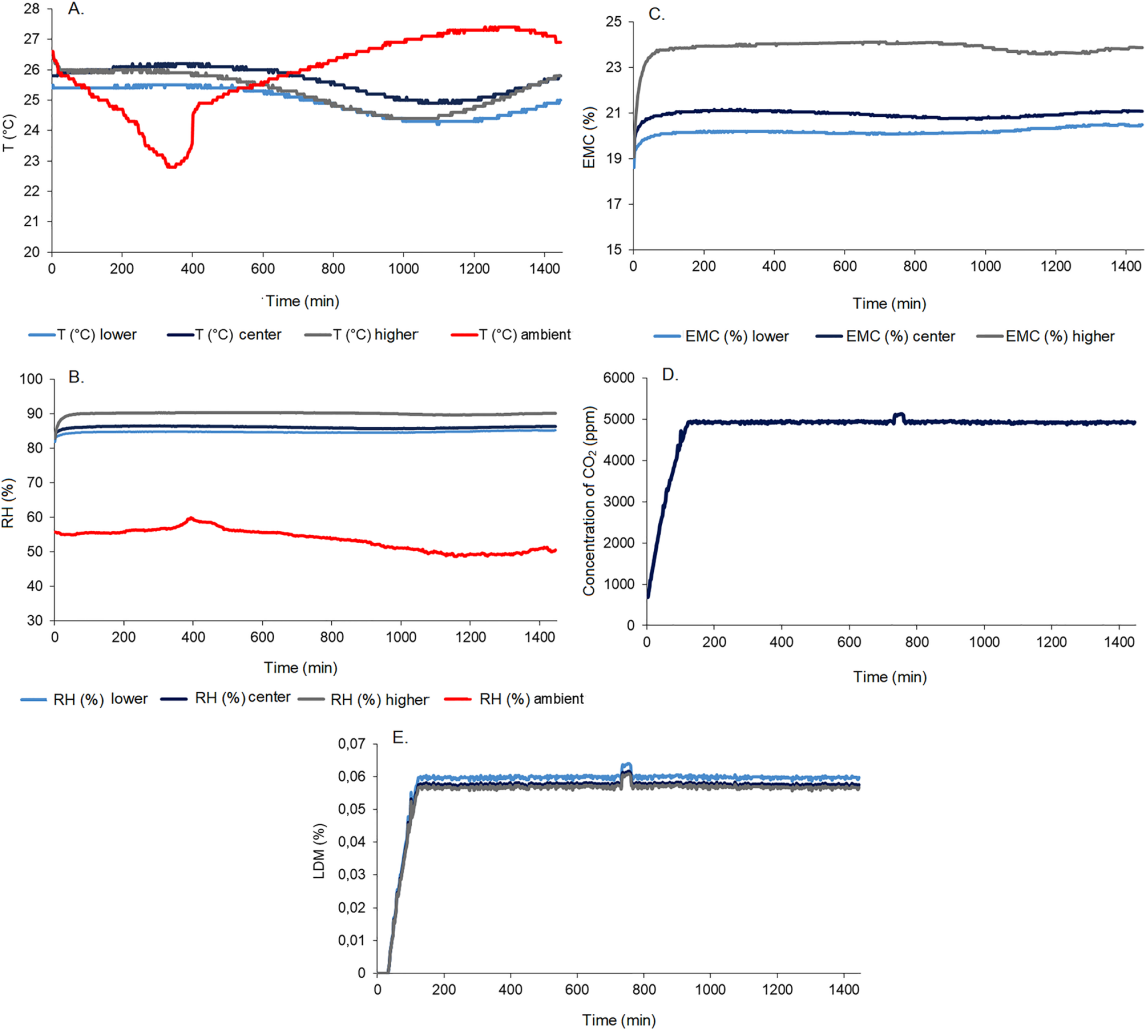
Monitoring temperature (**A**), relative humidity (**B**), equilibrium moisture content (**C**), carbon dioxide (**D**), loss of dry matter (**E**) of the intergranular air in the corn grain mass at 16% moisture content (w.b.) over time.

The findings reported by Bakhtavar et al.^29^ match the results for the lower and center grain layers, which suffered less influence from the external environment. Under these conditions, the average respiration of the grain mass was found to rise to 450 ppm in the first 3.5 h of monitoring (Fig. 4D), then reduced to the initial condition, remaining until the end of the monitoring period. As respiration levels remained low, with a CO_2_ concentration close to the ambient (420 ppm), there was no dry matter consumption in the grains. In the evaluation of the grain mass with moisture content of 16%, the same behavior of the curves of temperature and relative humidity of the intergranular air in the three layers (higher, center, and lower) was verified, even with variations in the external environment (Figs. 5A,B).

In this situation, the high moisture content (16%) had an influence on intergranular air gas exchange higher than the external condition (temperature and relative humidity). We observed that the temperature variation (Fig. 5A) was similar but higher (24.5–26.5 °C) than that observed in Fig. 4A. Meanwhile, the relative humidities in the three layers remained constant from the beginning to the end of the monitoring time (between 80 and 90%), being higher than the outside environment (between 50 and 60%) (Fig. 5B).

The conditions of 16% moisture content caused the grain mass to reach the equilibrium moisture content within the first hours of monitoring, remaining constant until the end of the period, between 19 and 21% in the lower and center layers and close to 24% in the top layer of the grain mass (Fig. 5C). Thus, in the first 2 h of monitoring, there was an intense respiration of the grain mass, reaching the limit of 5000 ppm of CO_2_ concentration and remaining constant until the final period of monitoring (Fig. 5D).

The high respiration of the grain mass provided a consumption and a loss of dry matter in the grains (Fig. 5E) during the first 2 h of monitoring. A 0.06% loss of dry matter was estimated over 24 h of monitoring, a significant result considering the short evaluation period^30^. Jian et al.^7^ evaluated interstitial carbon dioxide and oxygen concentrations in canola, soybean, and wheat seeds stored at different times, moisture contents, and temperatures. According to the authors, the greatest significant differences in CO_2_ concentrations were seen as a function of storage time and temperatures of 40 °C.

Ubhi and Sadaka^31^ found different respiration rates of corn grain mass as a function of temperatures of 23, 35 and 45 °C and 12.9, 14.8, 17.0, 18.8 and 20.7% initial moisture contents. The authors verified that the accumulated respiration reached 2.625 g/kg in grains with 18.8% moisture content and 35 °C average temperature after nine days of storage. Ochandio et al.^32^ reported a respiration rate of soybean seeds in airtight storage at 15, 25 and 35 °C and 13, 15 and 17% moisture contents from 0.130 to 20.272 mg CO_2_/(kg_dms_).

Coradi et al.^33^ evaluated the loss of dry matter of corn stored at 10 °C and 90%, 30 °C and 40% temperature and relative humidity, respectively. The authors found that corn stored at the 10 °C and 90% conditions had fungal contamination, whereas grain stored at 30 °C and 40% had a higher loss of dry matter and physical changes during storage time. Taher et al.^12^ built a model for predicting losses in soybeans stored in bag silo based on monitoring the CO_2_ concentration and verified through grain mass respiration losses of 0.07–2.16% of dry matter. Garcia-Cela et al.^34^ evaluated the respiration of naturally contaminated corn grains under different storage conditions at 0.80–0.99 water activity and 15–35 °C temperature. The highest respiration rate occurred at 0.95 water activity and temperature between 30 and 35 °C. Under these conditions, the authors found a higher loss of dry matter^35^.

### Evaluation of the physical quality of the grains

**Figure 6:**
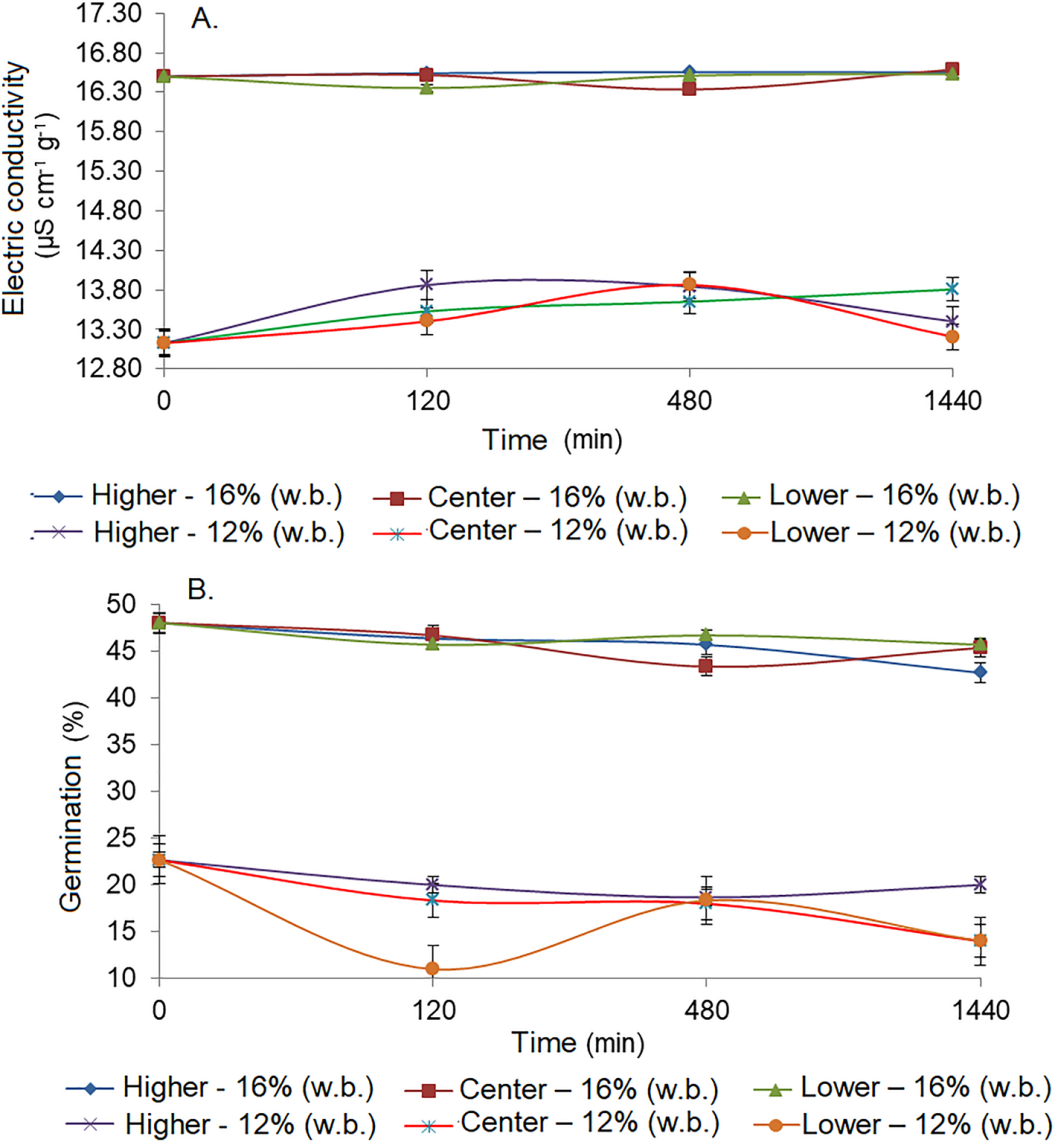
Evaluation of the cell structure of corn grains by the electrical conductivity test (**A**) and by the germination percentage (**B**) of corn grains with 12 and 16% moisture content (w.b.) over time.

Electrical conductivity test (Fig. 6A) indicated that the cellular structure of the corn grains was affected along the monitoring time as a function of the initial moisture contents (12 and 16%) and by the evaluation position (higher, center and lower layers) of the grain mass. Among the layers, we observed that the grains positioned in the upper (higher) layer suffered, slightly, the greatest physical changes, in agreement with the results obtained from the monitoring (temperature, relative humidity, and CO_2_). However, the greatest physical damages in the grains were found at a moisture content of 12%.

The electrical conductivity results reflected on the germination of the grains (Fig. 6B). The percentage of germinated grains was lower in the higher position of the grain layer and mainly for the grains with moisture contents of 12%. The increase in temperature, intergranular relative humidity, and hence, equilibrium moisture content, provided heating and a higher respiratory intensity of the grain mass, mainly for the grains with moisture contents of 16%. However, the increase of the metabolic activity of grains with 16% of moisture content (hydrated) within 24 h of monitoring, simulating a system of grain transport, collaborated to the increase of the percentage of germination when submitted to the test. It is noteworthy that the germination of grains is not desirable during transportation. In this case, the results obtained from germination are indicative of possible physical changes that can influence the final quality of the mass of grains.

Santos et al.^18^ evaluated the quality and loss of dry matter in corn grains stored at different temperatures. For this purpose, corn grains with initial moisture contents of 14.8 and 17.9% were packed in bags and stored at temperatures of 15, 25, and 35 °C. Over 150 days, at 30-day intervals, the concentrations of oxygen (O_2_) and carbon dioxide (CO_2_) were measured, and samples of the grains were taken for determining the moisture content, apparent specific mass, dry matter, germination percentage, and physical classification. In 150 days, the loss of dry matter in corn grains stored with a 14.8% moisture content was approximately 3.5 times lower than that for the product stored with a 17.9% moisture content. Based on the results of germination, a limit of 0.015% of loss of dry matter can be considered acceptable to maintain the integrity of the grains.

In a study carried out by Paraginski et al.^36^, the authors evaluated the quality of corn grains stored at temperatures of 5, 15, 25 and 35 °C for 12 months. The results of thousand-grain weight, germination, and electrical conductivity indicated that the greatest changes were observed in grains stored at the highest temperatures, mainly between 25 and 35 °C, indicating that the safe storage time of grains under these conditions should be shorter when compared to storage at low temperatures.

### Multivariate analysis

**Figure 7:**
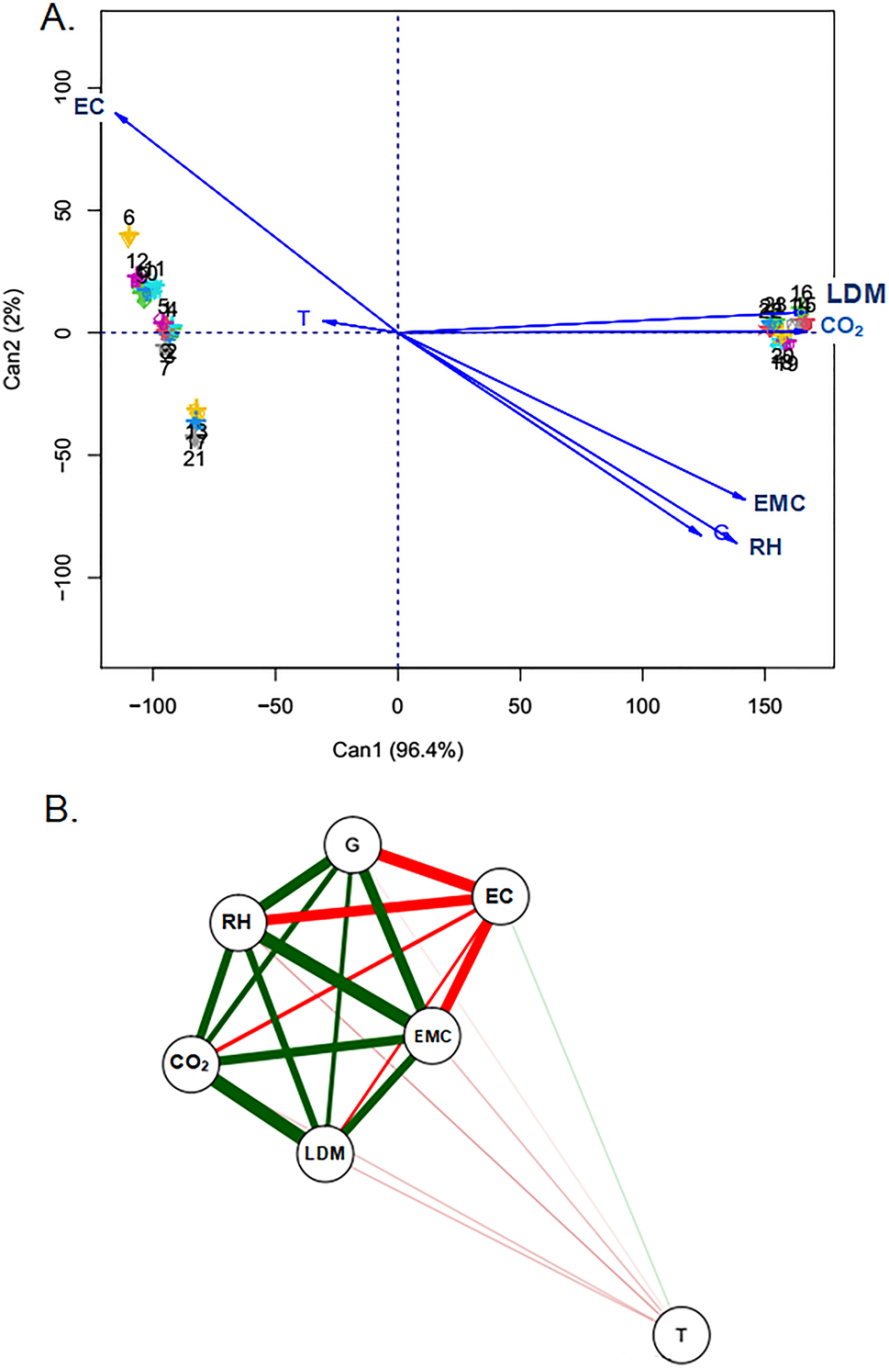
Analysis of the qualitative canonical variables of corn grains (**A**). Pearson correlation analysis of the qualitative variables of corn grains (**B**). Green lines link variables to positive correlation and red lines link negatively correlated variables. The thickness of the line is proportional to the correlation magnitude.

The analysis of the first two canonical variables gathered 98.4% of the total variation among treatments for the evaluated variables (Fig. 7A). In this biplot, treatments close to the Figure show high similarity. The vectors (arrows) point to the variables that most influenced the similarity of specific treatments.

When analyzing the quadrants, we verified the formation of three distinct groups, with clear patterns of associations with most of the variables evaluated. The first group allocated the treatments 8, 10, 11, 14, 15, 16, 18, 19, 20, 22, 23, and 24 that stood out in relation to the LDM and CO_2_ variables. The second group gathered the treatments 1, 2, 4, 5, 6, 7, 9, 12 with less variation in the variables T and EC. The third group contained treatments 13, 17 and 21, which did not stand out for any variable in a specific way. The variables RH, EMC and G did not stand out for any of the treatments, specifically.

From the clustering results, we verified that treatments with 16% moisture content at the top position of the grain layers and from the monitoring time of 2 h had the greatest influence on the variables, especially for carbon dioxide (CO_2_) and loss of dry matter (LDM). Barreto et al.^8^ developed a mathematical model to analyze grain quality by CO_2_ concentration using initial grain contents of 12, 14, and 16% (w.b.) and temperatures of 25 °C and 40 °C. For the 12% and 25 °C conditions, the CO_2_ concentration increased to 4%. For 16% moisture at 25 °C and 40 °C, the O_2_ decreased to less than 1%.

Correlation analysis between the variables are shown in Fig. 7B. A strong positive correlation can be seen between the variables LDM × CO_2_, EMC × RH, RH × G, and EMC × G, and a moderate positive correlation were shown between CO_2_ × RH, LDM × RH, CO_2_ × EMC, EMC × LDM and G × LDM. However, between the variables EC × G, EC × EMC, EC × RH, there was a strong negative correlation, with a moderate negative correlation shown between the variables EC × CO_2_. The variable T had a weak negative correlation with RH, EMC, LDM, CO_2_ and G, and a weak positive correlation with EC. The results observed in the correlation analysis are consistent with the behavior of the qualitative variables in relation to the treatment factors established for corn grain storage. The results corroborate those obtained by Coradi et al.^37^.

### Machine learning models

There were statistical differences (*p*-value < 0.01) between the evaluated machine learning techniques regarding the Pearson correlation coefficient (r) and mean absolute error (MAE) between the observed and estimated values for all the evaluated variables.

**Figure 8:**
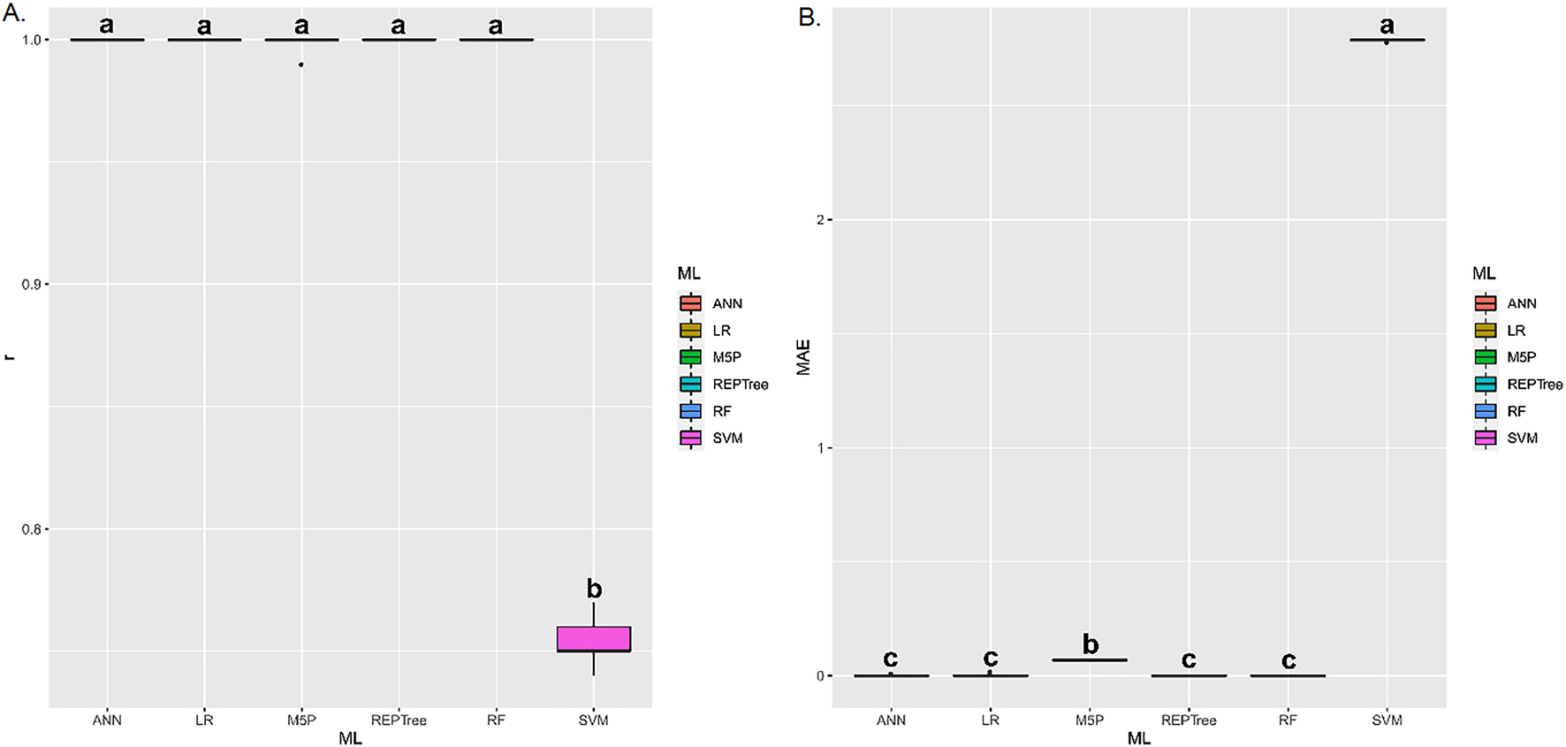
(**A**) Boxplot for Pearson correlation coefficient (r), and (**B**) mean absolute error (MAE) between observed and estimated values of apparent specific mass in corn grains by different machine learning models and inputs. Means followed by equal letters in the same column do not differ by the Scott–Knott test at 5% probability.

**Figure 9:**
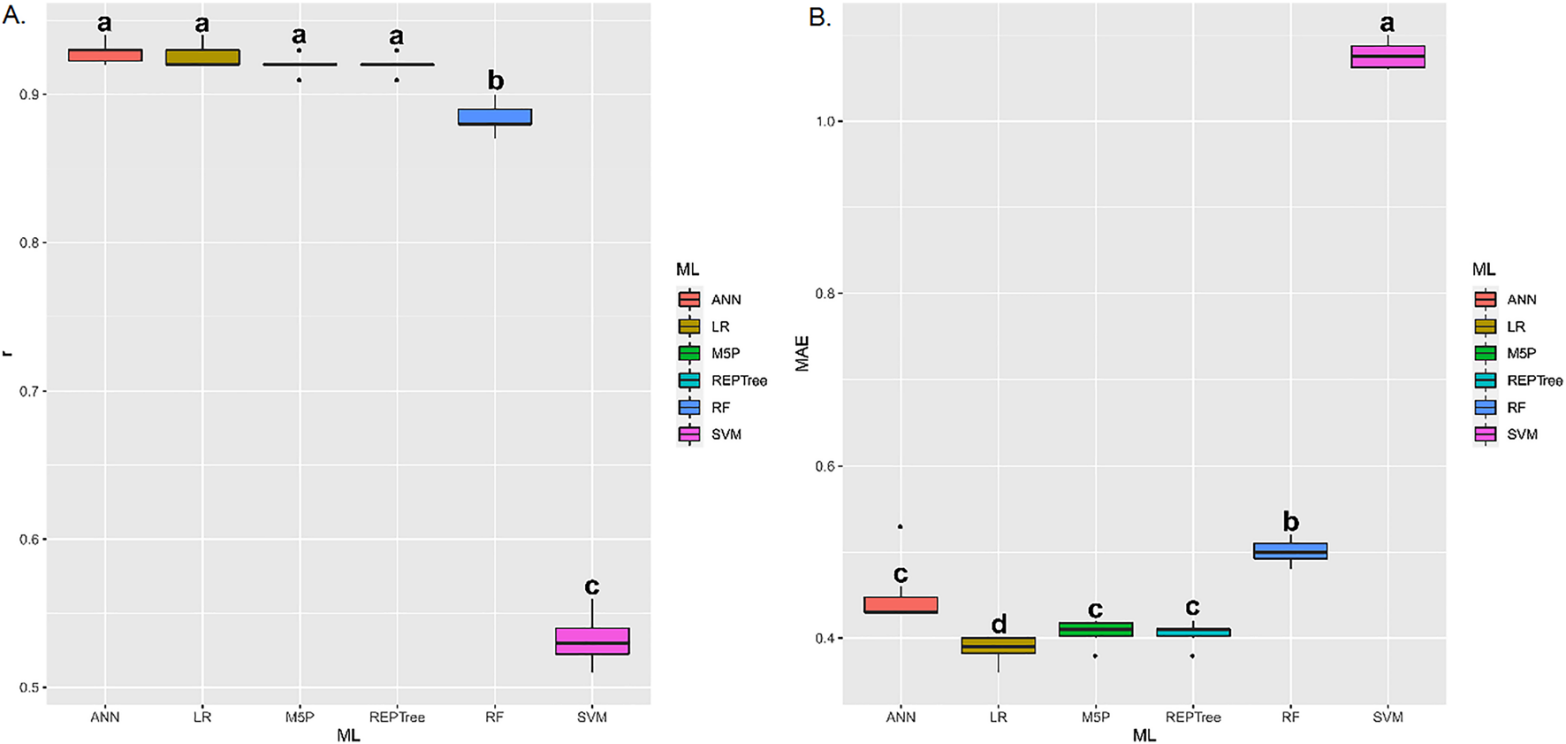
(**A**) Boxplot for Pearson correlation coefficient (r), and (**B**) mean absolute error (MAE) between observed and estimated values of electrical conductivity in corn grains by different machine learning models and inputs. Means followed by equal letters in the same column do not differ by the Scott–Knott test at 5% probability.

For apparent specific mass (Fig. 8A,B), the ANN, LR, M5P, REPTree, and RF techniques presented the highest r values, without statistically differing from each other. As for MAE, these same techniques, except M5P, presented the lowest averages. The ANN, LR, M5P, and REPTree techniques presented the highest r means between estimated and predicted values of electrical conductivity (Fig. 9A,B). However, when analyzing the MAE, it is observed that LR presented the lowest values.

**Figure 10:**
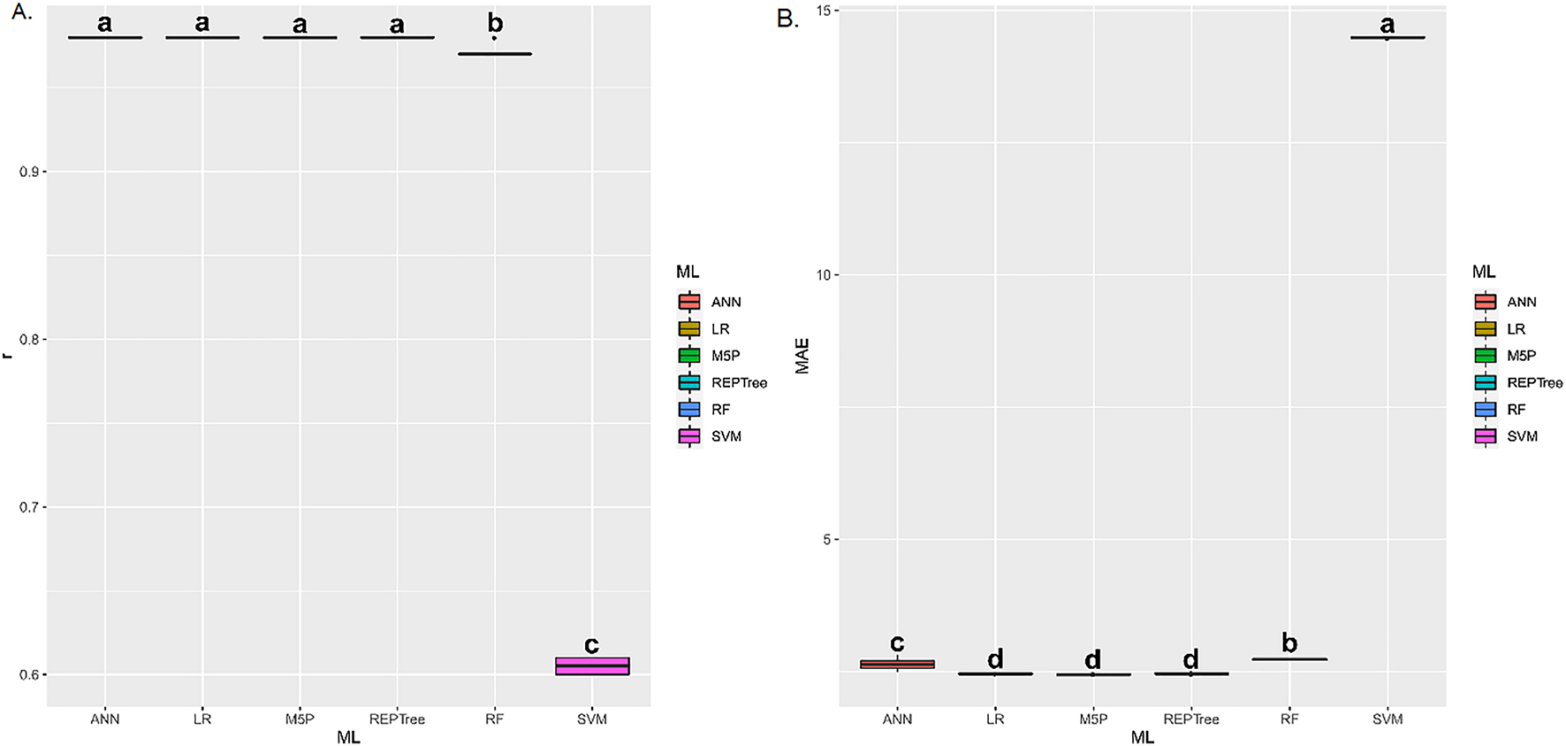
(**A**) Boxplot for Pearson correlation coefficient (r), and (**B**) mean absolute error (MAE) between observed and estimated values of germination in corn grains by different machine learning models and inputs. Means followed by equal letters in the same column do not differ by the Scott–Knott test at 5% probability.

**Figure 11:**
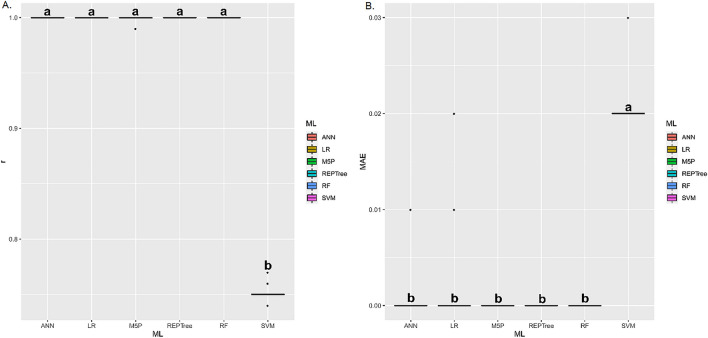
(**A**) Boxplot for Pearson correlation coefficient (r), and (**B**) mean absolute error (MAE) between observed and estimated values of loss of dry matter in corn grains by different machine learning models and inputs. Means followed by equal letters in the same column do not differ by the Scott-Knott test at 5% probability.

For germination (Fig. 10A,B), the ANN, LR, M5P, and REPTree techniques presented the highest r values, without statistically differing from each other. As for MAE, these same techniques, except ANN, presented the lowest means. The ANN, LR, M5P, REPTree, and RF techniques presented the highest r means between estimated and predicted values of electrical conductivity (Fig. 11A,B). These same techniques presented the lowest mean MAE.

## Conclusions

Real-time monitoring of the variable temperature, relative humidity, and carbon dioxide (CO_2_) concentration in the intergranular air determined early and satisfactorily in an indirect way the changes in the physical quality of the grains during transportation, confirmed by the physical analyses of electrical conductivity and germination.

In the first two hours of corn grain transport under the conditions of 16% moisture content in the top position of the grain mass profile suffered the highest physical quality changes, mainly regarding dry matter loss, due to the high equilibrium moisture content and respiration of the grain mass.

The application of Machine Learning predictive algorithms predicted the quantitative and qualitative losses of corn grains during transportation. All machine learning models, except support vector machine, obtained satisfactory results, equaling the multiple linear regression analysis.

## Supplementary Information

Supplementary Information 1.

Supplementary Information 2.

## Data Availability

All data generated or analysed during this study are included in this published article [and its supplementary information files: code programation and highlights]. The datasets used and/or analysed during the current study available from the corresponding author on reasonable request.

## References

[1] Conab. Monitoring the Brazilian grain harvest. <http://www.agricultura.gov.br>. (2019)

[2] Pereira PSX, Bianchini A, Caneppele C, Silva ARB, Machado RS, Pallaoro DS, Moraes FC (2019). Percentage of corn grain losses in roads transport based on weight of loads. J. Experim. Agric. Int..

[3] Coradi PC, Chaves JBP, Lacerda Filho AF, Mota TO (2014). Quality of stored grain of corn in different conditions. Científica.

[4] Lutz É, Coradi PC (2021). Applications of new technologies for monitoring and predicting grains quality stored: Sensors, internet of things, and artificial intelligence. Meas..

[5] Zhang SB, Zhai HC, Huang SX, Cai JP (2014). A site-directed CO_2_ detection method for monitoring the spoilage of stored grains by insects and fungi in Chinese horizontal warehouses. J. Stored Prod. Res..

[6] Coradi PC, Milane LV, Camilo LJ, Andrade MDO, Lima RE (2015). Quality of corn grain after drying and storage in natural environment and artificial cooling. Rev. Bras. Milho e Sorgo.

[7] Jian F, Chelladurai V, Jayas DS, Demianyk CJ, White NDG (2014). Interstitial concentrations of carbon dioxide and oxygen in stored canola, soybean, and wheat seeds under various conditions. J. Stored Prod. Res..

[8] Barreto AA, Abalone R, Gastón A, Ochandio D, Cardoso L, Bartosik R (2017). Validation of a heat, moisture and gas concentration transfer model for soybean (*Glycine max* L.) grains stored in plastic bags (silo bags). Biosyst. Eng..

[9] Coradi PC, Milane LV, Camilo LJ, Andrade MDO (2016). Drying and storage of corn grains for ethanol production in Brazil. Biosci. J..

[10] Huang, H., Danao, M. G., Rausch, K. & Singh, V. Diffusion and production of carbon dioxide in bulk corn at various temperatures and moisture content. In 2013 Kansas City, Missouri, July 21–July 24, (p. 1). American Society of Agricultural and Biological Engineers (2013).10.13031/aim.20131595312

[11] Patrício DI, Rieder R (2018). Computer vision and artificial intelligence in precision agriculture for grain crops: A systematic review. Comput. Electron. Agric..

[12] Taher HI, Urcola HA, Cendoya MG, Bartosik RE (2019). Predicting soybean losses using carbon dioxide monitoring during storage in silo bags. J. Stored Prod. Res..

[13] Besharati B, Lak A, Ghaffari H, Karimi H, Fattahzadeh M (2021). Development of a model to estimate moisture contents based on physical properties and capacitance of seeds. Sens. Actuators A..

[14] Jaques, L. B. A., Coradi, P. C., Lutz, É., Teodoro, P. E., Jaeger, D. V., & Teixeira, A. L. Nondestructive technology for real-time monitoring and prediction of soybean quality using Machine Learning for a bulk transport simulation. *IEEE Sens. J.* (2022).

[15] Coradi, P. C. & Lutz, E. SmartStorage—intelligent system for monitoring the quality of stored grains. Patent: Computer Program. Registration number: BR512022000638–3, registration date: 03/29/2022 (2022b)

[16] Jaques LBA, Coradi PC, Müller A, Rodrigues HE, Teodoro LPR, Teodoro PE, Steinhaus JI (2022). Portable-mechanical-sampler system for real-time monitoring and predicting soybean quality in the bulk transport. IEEE Trans. Instrum. Meas..

[17] ASAE. American Society of Agricultural Engineers. Moisture measurement unground grain and seeds. In: Standards, St. Joseph: ASAE, 563 (2000).

[18] Santos SBD, Martins MA, Faroni LRDA, Brito Junior VRD (2012). Dry matter loss in corn kernels stored in airtight bags. Rev. Cien. Agron..

[19] Brazil. Ministry of Agriculture, Livestock and Supply. Rules for Seed Analysis. Ministry of Agriculture, Livestock and Supply, Department of Agricultural Defense, Brasília, Mapa/ACS 399p. (2009).

[20] ISTA. International Seed Testing Association. Determination of other seeds by number. In: International rules for seed testing. ed. Bassersdorf, 41–43 (2008).

[21] Naldi MC, Campello RJ, Hruschka ER, Carvalho ACPLF (2011). Efficiency issues of evolutionary k-means. Appl. Soft Comput..

[22] Egmont-Petersen M, Ridder D, Handels H (2002). Image processing with neural networks-a review. Pattern Recognit..

[23] Blaifi SA, Moulahoum S, Benkercha R, Taghezouit B, Saim A (2018). M5P model tree based fast fuzzy maximum power point tracker. Sol. Energy.

[24] Kalmegh S (2015). Analysis of weka data mining algorithm reptree, simple cart and random tree for classification of indian news. Int. J. Innovat. Sci. Eng. Technol..

[25] Belgiu M, Dr ˘agu ¸t L (2016). Random forest in remote sensing: a review of applications and future directions. J. Photogramm. Remote Sens..

[26] Bouckaert, R. R., Frank, E., Hall, M., Kirkby, R., Reutemann, P., Seewald, A. & Scuse, D. WEKA manual for version 3–7–3. The University of Waikato 327 (2010).

[27] R Core Team. R: A language and environment for statistical computing. R Foundation for Statistical Computing, Vienna, Austria (2018).https://www.r-project.org/

[28] Lutz É, Coradi PC, Jaques LBA, Carneiro LO, Teodoro LPR, Teodoro PE, Souza GAC (2022). Real-time equilibrium moisture content monitoring to predict grain quality of corn stored in silo and raffia bags. J. Food Proc. Eng..

[29] Bakhtavar MA, Afzal I, Basra SMA (2019). Moisture adsorption isotherms and quality of seeds stored in conventional packaging materials and hermetic Super Bag. PLoS ONE.

[30] Coradi PC, Lutz É, Bilhalva NS, Jaques LBA, Leal MM, Teodoro LPR (2022). Prototype wireless sensor network and Internet of Things platform for real-time monitoring of intergranular equilibrium moisture content and predict the quality corn stored in silos bags. Expert Syst. Appl..

[31] Ubhi GS, Sadaka S (2015). Temporal valuation of corn respiration rates using pressure sensors. J. Stored Prod. Res..

[32] Ochandio D, Bartosik R, Gastón A, Abalone R, Barreto AA, Yommi A (2017). Modelling respiration rate of soybean seeds (Glycine max (L.)) in hermetic storage. J. Stored Prod. Res..

[33] Coradi PC, Lacerda Filho AF, Chaves JBP, Mota TO (2015). Loss of dry matter in grain corn stored under different conditions and effects on quality. Rev. Bras. Tecnol. Agroin..

[34] Garcia-Cela E, Kiaitsi E, Sulyok M, Krska R, Medina A, Petit Damico I, Magan N (2019). Influence of storage environment on maize grain: CO_2_ production, dry matter losses and aflatoxins contamination. Food Addit. Contam..

[35] Coradi PC, Lima RE, Alves CZ, Teodoro PE, Cândido ACDS (2020). Evaluation of coatings for application in raffia big bags in conditioned storage of soybean cultivars in seed processing units. PLoS ONE.

[36] Paraginski RT, Rockenbach BA, Santos RFD, Elias MC, Oliveira MD (2015). Quality of corn grains stored at different temperatures. Rev. Bras. Eng. Agric. Amb..

[37] Coradi PC, Padia CL, Jaques LBA, Souza GACD, Lima RE, Müller A, Teodoro PE, Steinhaus JI, Carneiro LDO (2020). Adaptation of technological packaging for conservation of soybean seeds in storage units as an alternative to modified atmospheres. PLoS ONE.

