# Supplementary material for: Sensor-cable-probe and sampler for early detection and prediction of dry matter loss and real-time corn grain quality in transport and storage
Source: Sci Rep. 2023 Apr 7;13:5686. doi: 10.1038/s41598-023-32684-4 (PMC10082028; doi:10.1038/s41598-023-32684-4)
Supplement: Supplementary file 1 — Supplementary Information 1. [file 41598_2023_32684_MOESM1_ESM.doc]

**Code programation**

/////////////////////////////////////// DHT22 //////////////////////////////////

#include <Adafruit_Sensor.h>

#include <DHT.h>

#include <DHT_U.h>

#include <math.h>

#define DHTTYPE DHT22

#define DHTPIN 2

#define DHTPIN1 3

#define DHTPIN2 7

DHT_Unified dht(DHTPIN, DHTTYPE);

DHT_Unified dht1(DHTPIN1, DHTTYPE);

DHT_Unified dht2(DHTPIN2, DHTTYPE);

float umid = 0, temp = 0, umid1= 0, temp1 = 0,umid2 = 0, temp2 = 0;

float base = 2.718281828, expoente = 0.899, c= -0.708141, b= 0.004893, t = 1, a= -0.212369;

double resultado0, resultado1, resultado2, resultado3, resultado4;

/*float valores[6];

valores[0]=temp;

valores[1]=temp1;

valores[2]=temp2;

valores[3]=umid;

valores[4]=umid1;

valores[5]=umid2;*/

////////////////////////////////// SD card /////////////////////////////////////

#include <SD.h> //Load SD library

int chipSelect = 4; //chip select pin for the MicroSD Card Adapter

File file; // file object that is used to read and write data

///////////////////////////////// RTC //////////////////////////////////////////

#include "Wire.h"

#define DS3231_I2C_ADDRESS 0x68

// Convert normal decimal numbers to binary coded decimal

byte decToBcd(byte val)

{

return( (val/10*16) + (val%10) );

}

// Convert binary coded decimal to normal decimal numbers

byte bcdToDec(byte val)

{

return( (val/16*10) + (val%16) );

}

///////////////////////////////// CO2 //////////////////////////////////////////

#include <SoftwareSerial.h>

const int analogPin = A0;

const int pwmPin = 9;

const long samplePeriod = 10000L;

SoftwareSerial sensor(10, 11); // TX, RX

const byte requestReading[] = {0xFF, 0x01, 0x86, 0x00, 0x00, 0x00, 0x00, 0x00, 0x79};

byte result[9];

long lastSampleTime = 0;

/////////////////////////////////////////////////////////////////////////////////////

void setup() {

Serial.begin(9600); // start serial connection to print out debug messages and data

/////////////////////////////// CO2 //////////////////////////////////////////

sensor.begin(9600);

pinMode(pwmPin, INPUT_PULLUP);

/////////////////////////////// SD card //////////////////////////////////////

pinMode(chipSelect, OUTPUT); // chip select pin must be set to OUTPUT mode

if (!SD.begin(chipSelect))

{ // Initialize SD card

Serial.println("Could not initialize SD card."); // if return value is false, something went wrong.

}

if (SD.exists("file.txt"))

{ // if "file.txt" exists, fill will be deleted

Serial.println("File exists.");

if (SD.remove("file.txt") == true)

{

Serial.println("Successfully removed file.");

}

else

{

Serial.println("Could not removed file.");

}

}

?

///////////////////////////////////// DHT22 ////////////////////////////////////

dht.begin(); // inicializa a função

dht1.begin();

dht2.begin();

////////////////////////////////// RTC ///////////////////////////////////////

Wire.begin();

// set the initial time here:

// DS3231 seconds, minutes, hours, day, date, month, year

//setDS3231time(00,35,15,05,13,02,20);

//////////////////////////////////////////////////////////////////////////////

}

void loop()

{

////////////////////////////////// CO2 ///////////////////////////////////////

long now = millis();

int ppmV = readPPMV();

int ppmPWM = readPPMPWM();

if (now > lastSampleTime + samplePeriod)

{

lastSampleTime = now;

int ppmV = readPPMV();

//int ppmS = readPPMSerial();

int ppmPWM = readPPMPWM();

//Serial.print(ppmV);

//Serial.print("\t");

//Serial.print(ppmPWM);

//Serial.print("\t");

//Serial.println(ppmS);

}

///////////////////////////// DHT22 //////////////////////////////////////////

sensors_event_t event;

dht.temperature().getEvent(&event);

temp = event.temperature;

dht.humidity().getEvent(&event);

umid = event.relative_humidity;

dht1.temperature().getEvent(&event);

temp1 = event.temperature;

dht1.humidity().getEvent(&event);

umid1 = event.relative_humidity;

dht2.temperature().getEvent(&event);

temp2 = event.temperature;

dht2.humidity().getEvent(&event);

umid2 = event.relative_humidity;

/*Serial.print("Temperatura Sensor 1: ");

Serial.print(temp);

Serial.print("C");

Serial.print(" Umidade Sensor 1: ");

Serial.print(umid);

Serial.println("%");

delay(1000);

Serial.print("Temperatura Sensor 2: ");

Serial.print(temp1);

Serial.print("C");

Serial.print(" Umidade Sensor 2: ");

Serial.print(umid1);

Serial.println("%");

delay(1000);

Serial.print("Temperatura Sensor 3: ");

Serial.print(temp2);

Serial.print("C");

Serial.print(" Umidade Sensor 3: ");

Serial.print(umid2);

Serial.println("%");

Serial.println("");

delay(5000);*/

////////////////////////////////// SD card ///////////////////////////////////

float base = 2.718281828, expoente = (umid/100), c= -0.708141, b= 0.004893, t = temp, a= -0.212369;

float expoente1= (umid1/100), t1= temp1;

float expoente2= (umid2/100), t2= temp2;

double resultado0, resultado1, resultado2, resultado3, resultado4;

resultado0 = pow(base, expoente);

resultado1 = resultado0*c;

resultado2 = b*t;

resultado3= a - resultado2 + resultado1;

resultado4= pow(base,resultado3);

double resultado5, resultado6, resultado7, resultado8, resultado9;

resultado5 = pow(base, expoente1);

resultado6 = resultado5*c;

resultado7 = b*t1;

resultado8= a - resultado7 + resultado6;

resultado9= pow(base,resultado8);

double resultado10, resultado11, resultado12, resultado13, resultado14;

resultado10 = pow(base, expoente2);

resultado11 = resultado10*c;

resultado12 = b*t2;

resultado13= a - resultado12 + resultado11;

resultado14= pow(base,resultado13);

file = SD.open("file.txt", FILE_WRITE); // open "file.txt" to write data

if (file)

{

//int number = random(10); // generate random number between 0 and 9

//float valores[6]={umid, temp, umid1, temp1, umid2, temp2};

//file.println(valores); // write number to file

//float number[6] = {umid, temp, umid1, temp1, umid2, temp2};

file.print(temp); // write number to file

file.print("\t"); // write number to file

file.print(temp1); // write number to file

file.print("\t"); // write number to file

file.print(temp2); // write number to file

file.print("\t"); // write number to file

file.print(umid); // write number to file

file.print("\t"); // write number to file

file.print(umid1); // write number to file

file.print("\t"); // write number to file

file.print(umid2); // write number to file

file.print("\t"); // write number to file

file.print(ppmV); // write number to file

file.print("\t"); // write number to file

file.print(ppmPWM); // write number to file

file.print("\t"); // write number to file

file.print(resultado4); // write number to file

file.print("\t"); // write number to file

file.print(resultado9); // write number to file

file.print("\t"); // write number to file

file.print(resultado14); // write number to file

file.print("\t"); // write number to file

displayTime();

file.print("\n"); // write number to file

file.close(); // close file

Serial.print("\n"); // write number to file

Serial.println("Wrote number: ");

Serial.print(temp);

Serial.print("\t"); // write number to file

Serial.print(temp1);

Serial.print("\t"); // write number to file

Serial.print(temp2);

Serial.print("\t"); // write number to file

Serial.print(umid);

Serial.print("\t"); // write number to file

Serial.print(umid1);

Serial.print("\t"); // write number to file

Serial.print(umid2);

Serial.print("\t"); // write number to file

Serial.print(ppmV);

Serial.print("\t"); // write number to file

Serial.print("\n"); // write number to file

Serial.print(ppmPWM);

Serial.print("\t"); // write number to file

Serial.print("\n"); // write number to file

Serial.print(resultado4);

Serial.print("\t"); // write number to file

Serial.print("\n"); // write number to file

Serial.print(resultado9);

Serial.print("\t"); // write number to file

Serial.print("\n"); // write number to file

Serial.print(resultado14);

Serial.print("\t"); // write number to file

Serial.print("\n"); // write number to file

}

else

{

Serial.println("Could not open file (writing).");

}

file = SD.open("file.txt", FILE_READ);

{

Serial.println("--- Reading start ---");

char character;

while ((character = file.read()) != -1)

{ Serial.print(character);

}

file.close();

Serial.println("--- Reading end ---");

}

else

{

Serial.println("Could not open file (reading).");

}

delay(50000); // wait for 50000ms

/////////////////////////////////// RTC /////////////////////////////////////

displayTime(); // display the real-time clock data on the Serial Monitor

delay(50000); // every second

}

////////////////////////////////////// RTC /////////////////////////////////////

/*void setDS3231time(byte second, byte minute, byte hour, byte dayOfWeek, byte

dayOfMonth, byte month, byte year)

{

// sets time and date data to DS3231

Wire.beginTransmission(DS3231_I2C_ADDRESS);

Wire.write(0); // set next input to start at the seconds register

Wire.write(decToBcd(second)); // set seconds

Wire.write(decToBcd(minute)); // set minutes

Wire.write(decToBcd(hour)); // set hours

Wire.write(decToBcd(dayOfWeek)); // set day of week (1=Sunday, 7=Saturday)

Wire.write(decToBcd(dayOfMonth)); // set date (1 to 31)

Wire.write(decToBcd(month)); // set month

Wire.write(decToBcd(year)); // set year (0 to 99)

Wire.endTransmission();

}*/

void readDS3231time(byte *second,

byte *minute,

byte *hour,

byte *dayOfWeek,

byte *dayOfMonth,

byte *month,

byte *year)

{

Wire.beginTransmission(DS3231_I2C_ADDRESS);

Wire.write(0); // set DS3231 register pointer to 00h

Wire.endTransmission();

Wire.requestFrom(DS3231_I2C_ADDRESS, 7);

// request seven bytes of data from DS3231 starting from register 00h

*second = bcdToDec(Wire.read() & 0x7f);

*minute = bcdToDec(Wire.read());

*hour = bcdToDec(Wire.read() & 0x3f);

*dayOfWeek = bcdToDec(Wire.read());

*dayOfMonth = bcdToDec(Wire.read());

*month = bcdToDec(Wire.read());

*year = bcdToDec(Wire.read());

}

void displayTime()

{

byte second, minute, hour, dayOfWeek, dayOfMonth, month, year;

// retrieve data from DS3231

readDS3231time(&second, &minute, &hour, &dayOfWeek, &dayOfMonth, &month,

&year);

// send it to the serial monitor

Serial.print(hour, DEC);

file.print(hour, DEC);

Serial.print(":");

file.print(":"); // write number to file

if (minute<10)

{

Serial.print("0");

file.print("0"); // write number to file

}

Serial.print(minute, DEC);

file.print(minute, DEC);

Serial.print(":");

file.print(":");

if (second<10)

{

Serial.print("0");

file.print("0");

}

Serial.print(second, DEC);

file.print(second, DEC);

Serial.print(" ");

file.print(" ");

Serial.print(dayOfMonth, DEC);

file.print(dayOfMonth, DEC);

Serial.print("/");

file.print("/");

Serial.print(month, DEC);

file.print(month, DEC);

Serial.print("/");

file.print("/");

Serial.print(year, DEC);

file.print(year, DEC);

/* Serial.print(" Day of week: ");

file.print(" Day of week: ");

switch(dayOfWeek){

case 1:

Serial.println("Sunday");

file.print("Sunday");

break;

case 2:

Serial.println("Monday");

file.print("Monday");

break;

case 3:

Serial.println("Tuesday");

file.print("Tuesday");

break;

case 4:

Serial.println("Wednesday");

file.print("Wednesday");

break;

case 5:

Serial.println("Thursday");

file.print("Thursday");

break;

case 6:

Serial.println("Friday");

file.print("Friday");

break;

case 7:

Serial.println("Saturday");

file.print("Saturday");

break;

}*/

Serial.print("\n");

//file.print("\n");

}

////////////////////////////////// CO2 /////////////////////////////////////////

int readPPMV()

{

float v = analogRead(analogPin) * 5.0 / 1023.0;

int ppm = int((v - 0.4) * 3125.0);

return ppm;

}

/*int readPPMSerial()

{

for (int i = 0; i < 9; i++)

{

sensor.write(requestReading[i]);

}

//Serial.println("sent request");

while (sensor.available() < 9) {};

for (int i = 0; i < 9; i++)

{

result[i] = sensor.read();

}

int high = result[2];

int low = result[3];

//Serial.print(high); Serial.print(" ");Serial.println(low);

return high * 256 + low;

}*/

int readPPMPWM()

{

while (digitalRead(pwmPin) == LOW) {};

long t0 = millis();

while (digitalRead(pwmPin) == HIGH) {};

long t1 = millis();

while (digitalRead(pwmPin) == LOW) {};

long t2 = millis();

long th = t1-t0;

long tl = t2-t1;

long ppm = 5000L * (th - 2) / (th + tl - 4);

while (digitalRead(pwmPin) == HIGH) {};

delay(10);

return int(ppm);

}
