# Supplementary material for: Sensor-cable-probe and sampler for early detection and prediction of dry matter loss and real-time corn grain quality in transport and storage
Source: Sci Rep. 2023 Apr 7;13:5686. doi: 10.1038/s41598-023-32684-4 (PMC10082028; doi:10.1038/s41598-023-32684-4)
Supplement: Supplementary file 2 — Supplementary Information 2. [file 41598_2023_32684_MOESM2_ESM.doc]

**Highlights**

- Non-destructive technology for real-time monitoring of corn quality on the bulk transport.
- Monitoring of equilibrium moisture content and carbon dioxide to prevent corn transport losses.
- Predicting the corn quality on the bulk transport using Machine Learning.
- Early detection dry matter loss of corn grains in real time during transport.
